# Comparison of conventional mechanical ventilation and high-frequency oscillatory ventilation in congenital diaphragmatic hernias: a systematic review and meta-analysis

**DOI:** 10.1038/s41598-023-42344-2

**Published:** 2023-09-26

**Authors:** Hee-Beom Yang, Agostino Pierro, Hyun-Young Kim

**Affiliations:** 1 Department of Surgery, Seoul National University Bundang Hospital, Seongnam, South Korea; 2 Department of Surgery, College of Medicine, Seoul National University, Seoul, South Korea; 3 Translational Medicine, Research Institute, Hospital for Sick Children, Toronto, Canada; 4 Division of General and Thoracic Surgery, The Hospital for Sick Children, Toronto, Canada; 5 Department of Pediatric Surgery, Seoul National University Children’s Hospital, Seoul, South Korea

**Keywords:** Neonatology, Respiratory signs and symptoms

## Abstract

Outcomes of conventional mechanical ventilation (CMV) and high-frequency oscillatory ventilation (HFOV) in patients with congenital diaphragmatic hernia (CDH) were compared through a systematic review and meta-analysis. Outcome measures included mortality and incidence of chronic lung disease (CLD). Odds ratio (OR) and 95% confidence interval (95%CI) were evaluated. Subgroup analyses were performed according to the strategy for applying HFOV in CDH patients. Group A: CMV was initially applied in all CDH patients, and HFOV was applied in unstable patients. Group B: chronologically analyzed. (CMV and HFOV era) Group C: CMV or HFOV was used as the initial MV. Of the 2199 abstracts screened, 15 full-text articles were analyzed. Regarding mortality, 16.7% (365/2180) and 32.8% (456/1389) patients died in CMV and HFOV, respectively (OR, 2.53; 95%CI 2.12–3.01). Subgroup analyses showed significantly worse, better, and equivalent mortality for HFOV than that for CMV in group A, B, and C, respectively. CLD occurred in 32.4% (399/1230) and 49.3% (369/749) patients in CMV and HFOV, respectively (OR, 2.37; 95%CI 1.93–2.90). The evidence from the literature is poor. Mortality and the incidence of CLD appear worse after HFOV in children with CDH. Cautious interpretation is needed due to the heterogeneity of each study.

## Introduction

Congenital diaphragmatic hernia (CDH) is a rare, life-threatening malformation characterized by diaphragmatic defect and herniation of abdominal organs into the thoracic cavity with its estimated prevalence of 2.3 per 10,000 live births^1,2^. Causes of mortality for CDH are mainly pulmonary hypoplasia and pulmonary hypertension. Endeavors to improve its mortality include immediate resuscitation, adequate mechanical ventilation, and cardiac support in case of severe cardiac dysfunction^2^. High-frequency oscillatory ventilation (HFOV) can be an elective rescue mode of ventilation as indicated by the APSA outcomes and evidence-based practice committee^3^. Therefore, the VICI-trial was conducted to determine the optimal initial ventilation mode in congenital diaphragmatic hernia^4^. Yet there is lack of evidence regarding the comparison of incidence and mortality for chronic lung disease (CLD) between conventional mechanical ventilation (CMV) and HFOV in CDH patients. Hence, we performed this systematic review and meta-analysis to compare these outcomes in CDH.

## Materials and methods

### Protocol and registration

This systematic review and meta-analysis adhered to the Preferred Reported Items for Systematic Reviews and Meta-Analysis (PRISMA) guidelines. The protocol was registered in PROSPERO (registration number: CRD42022325445).

### Literature search

The literature search was performed using PubMed, EMBASE (Ovid), Cochrane library, Web of Science, and Cumulative Index to Nursing and Allied Health Literature (CINAHL) databases. The following keywords were used: congenital diaphragmatic hernia, mechanical ventilation, and high-frequency ventilation. The detailed search strategy is presented in the supplemental material. The search was conducted in April 2022.

### Eligibility criteria

Any study reporting comparison of mortality and the incidence of CLD between CMV versus HFOV in CDH was considered eligible. Language was restricted to English. Case reports, review articles, letters, and congress abstracts were excluded. Inclusion criteria were studies which reported the outcome (incidence and mortality of CLD) after comparing CMV and HFOV in infants with CDH. Studies without this comparison were excluded.

### Study selection and methodological quality assessment

Two reviewers (H.-B. Y. and H.-Y. K.) independently screened title and abstract of the studies according to the criteria listed above, among the studies retrieved using the search strategy. After first screening, full text was reviewed for final inclusion. These two investigators independently assessed the quality and came to a consensus of all papers that met our inclusion criteria using the Newcastle–Ottawa Scale for comparative studies. Joint review was performed in case of inconsistency.

### Data extraction

Systematical extraction of the data was performed regarding study design, study period, mortality, subgroup, and the incidence of CLD from the included studies. A data extraction form was used to record the results.

### Subgroup analysis

All studies included in this meta-analysis compared the outcomes of CMV and HFOV. However, heterogeneity was observed regarding the strategy for applying HFOV. Some studies applied HFOV in selected patients who showed persistent unstable vital signs after being initially ventilated with CMV. Others applied HFOV at the first timing of MV and selected either CMV or HFOV. In these studies, CMV was not routinely used as the initial MV strategy. The others compared the historical outcomes of CMV and HFOV. The outcomes of CMV before introducing HFOV were compared with the outcomes of HFOV. Herein, subgroup analyses were performed according to the strategy for applying HFOV in CDH patients. In group A, CMV was initially applied in all CDH patients, and HFOV was applied in unstable patients showing poor outcomes in CMV. In group B, the results of CMV and HFOV were chronologically analyzed. The use of HFOV was more recent than the use of CMV in all studies in group B. In group C, either CMV or HFOV was used as the initial MV, which is different from group A, which used CMV as the routine initial MV strategy.

### Statistical analysis

Statistical analysis was performed using R × 64 4.1.2. Pooled estimates of odds ratios (ORs) with 95% confidence intervals (95%CIs) were evaluated using standard methods. Both fixed and random-effects models were used. Heterogeneity between studies was assessed using the I^2^ statistic. The forest plot and funnel plot were used to estimate the overall result and publication bias, respectively.

## Results

**Figure 1:**
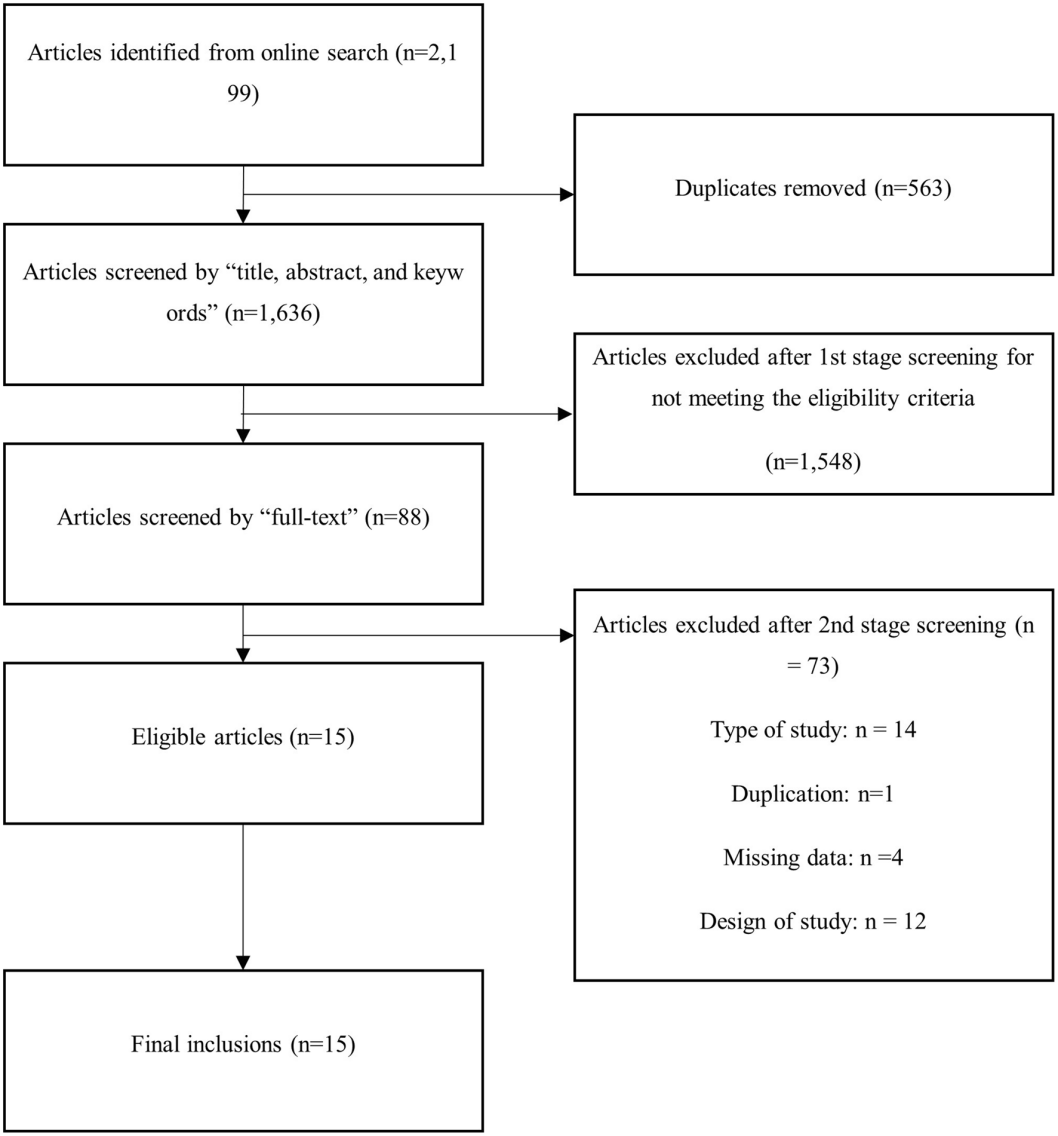
Diagram of workflow in the systematic review.

Of 2199 studies found by defined search strategy, 1636 abstracts were reviewed, and 88 full-text articles were examined. Of these, 15 studies were included in this study^4–18^ (Fig. 1). There was one prospective randomized clinical trial. Table 1 summarizes the presentation center, patient enroll timing, and main results of the study. Mortality rate were reported in all studies and the incidence of CLD was reported in 6 papers among the 15 included studies. Various countries, including the United Kingdom, the United States, France, and Canada conducted the studies. Patients were included from 1981 to 2017. Most of them were retrospective studies and one paper was prospective RCT.

**Table 1:** Overall results of meta-analysis comparing conventional mechanical ventilation and high-frequency oscillatory ventilation.

Ref.	Mortality	CLD	Center	Period of recruitment	Prospective/retrospective	Timing of CMV//HFOV application	Death/total cases		CLD/total cases	
							**CMV**	**HFOV**	**CMV**	**HFOV**
^5^	O		Indigra Gandhi Institute Of Child Health, India	2005–2017	Retrospective	HFOV when unstable after initial CMV	1/66	2/7		
^6^	O		The Hospital for Sick Children, Canada	1981–1984	Retrospective	HFOV when unstable after initial CMV	27/136	74/87		
^7^	O		The Montreal Children's Hospital, Canada	2005–2010	Retrospective	HFOV when unstable after initial CMV	13/248	8/46		
^8^	O		Orsola-Malpighi Hospital, Italy	1987–1997	Retrospective	Certain period CMV or HFOV	11/25	4/19		
^9^	O	O	Child Health and Children’s Hospital Research Institute of Manitoba, Canada	1991–2015	Retrospective	Initially CMV or HFOV	0/41	5/39	5/41	16/34
^10^	O		UFR Cochin-Port Royal, France	1985–1997	Retrospective	Certain period CMV or HFOV	14/19	11/32		
^11^	O	O	Japanese Congenital Diaphragmatic Hernia Study Group	2011–2016	Retrospective	HFOV when unstable after initial CMV	2/77	39/250	19/72	94/210
^12^	O		The Children’s Hospital at Westmead, Australia	2003–2018	Retrospective	HFOV when unstable after initial CMV	23/120	17/39		
^13^	O	O	St Mary’s Hospital, UK	1991–2005	Retrospective	Certain period CMV or HFOV	13/21	12/44	5/11	9/30
^14^	O		King’s College Hospital, UK	2011–2015	Retrospective	HFOV when unstable after initial CMV	4/25	8/14		
^15^	O		UC Irvine Medical Center, USA	1993–1996	Retrospective	HFOV when unstable after initial CMV	0/3	4/18		
^16^	O		All India Institute of Medical Sciences, India	2014–2017	Retrospective	HFOV when unstable after initial CMV	7/17	15/20		
^4^	O	O	CDH EURO Consortium	2008–2013	Prospective	Initially CMV or HFOV	21/91	25/80	21/70	18/55
^17^	O	O	University of Tsukuba	1991–2011	Retrospective	Certain period CMV or HFOV	11/29	1/20	2/21	3/19
^18^	O	O	Congenital Diaphragmatic Hernia Study Group	2001–2006	Retrospective	HFOV when unstable after initial CMV	218/1262	231/674	346/1015	229/401

### Mortality

**Figure 2:**
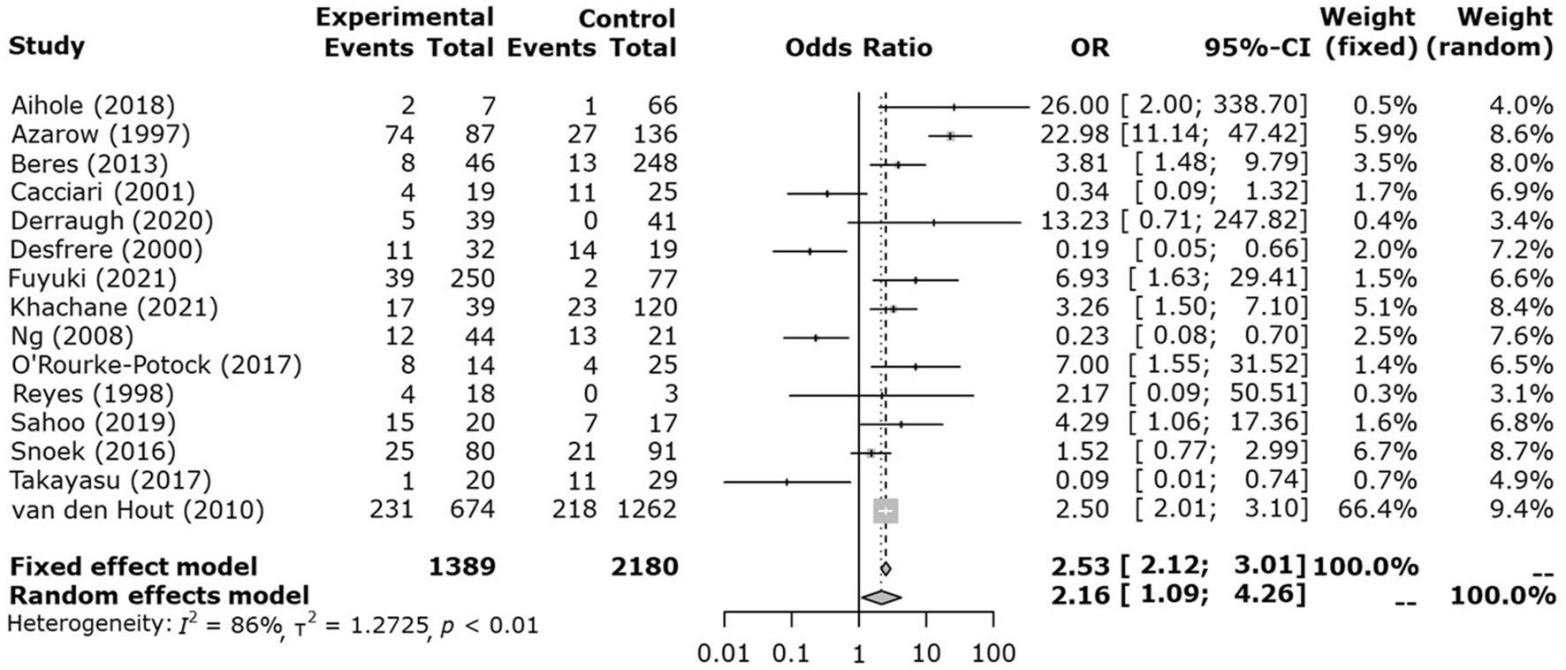
Forest plot of overall mortality.

The pooled result of all enrolled studies for mortality showed significantly higher mortality in HFOV (Fig. 2). In total, there were 456 deaths in HFOV (n = 1389) and 365 deaths in CMV (n = 2180). The ORs were 2.53 for the fixed model and 2.16 for the random-effect model, respectively; 95%CIs did not contain the value 1. In I^2^ statistics, heterogeneity was observed to be 86%, higher than the 50% cut-off, indicating high heterogeneity between studies.

**Figure 3:**
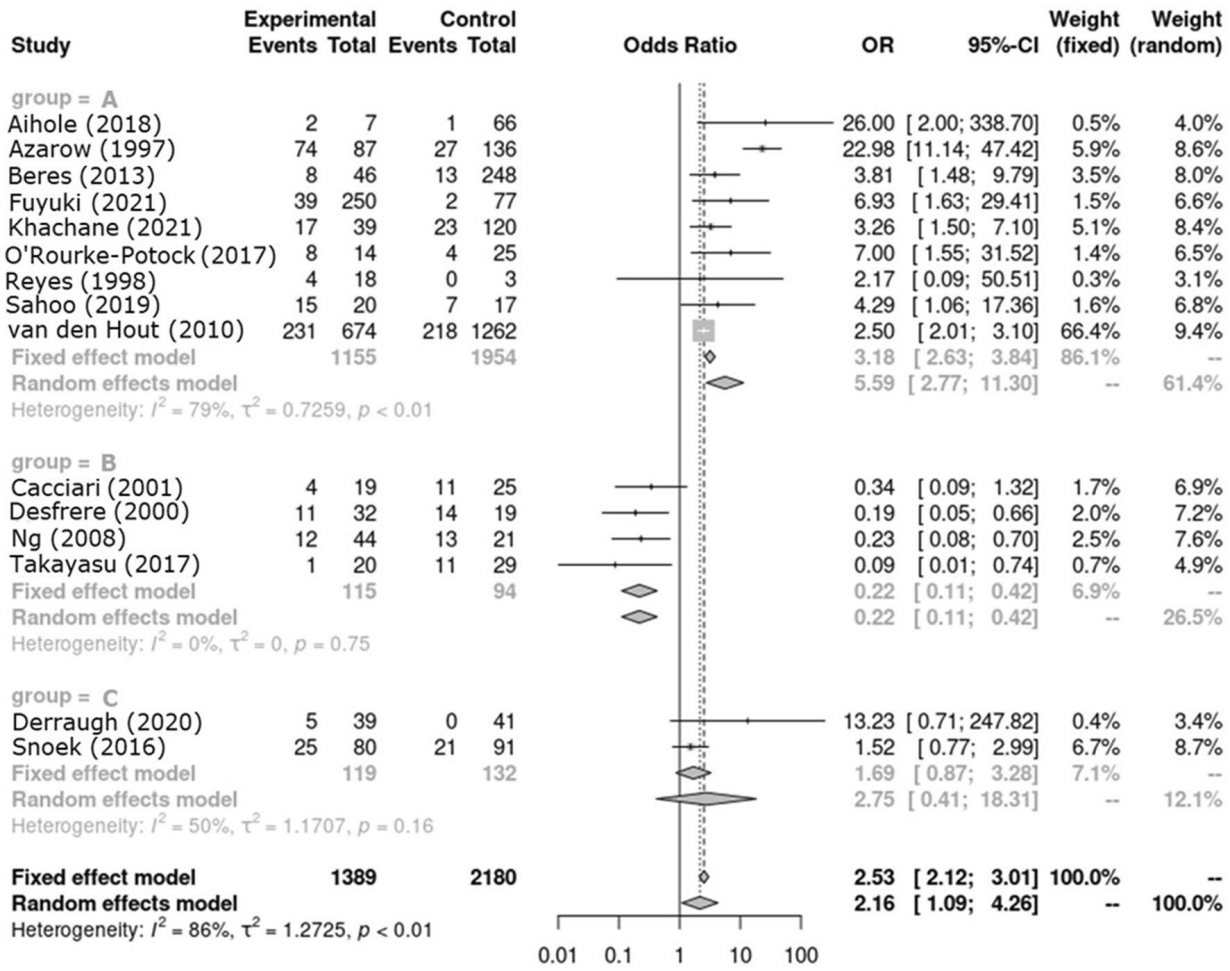
Forest plot of subgroup mortality.

**Figure 4:**
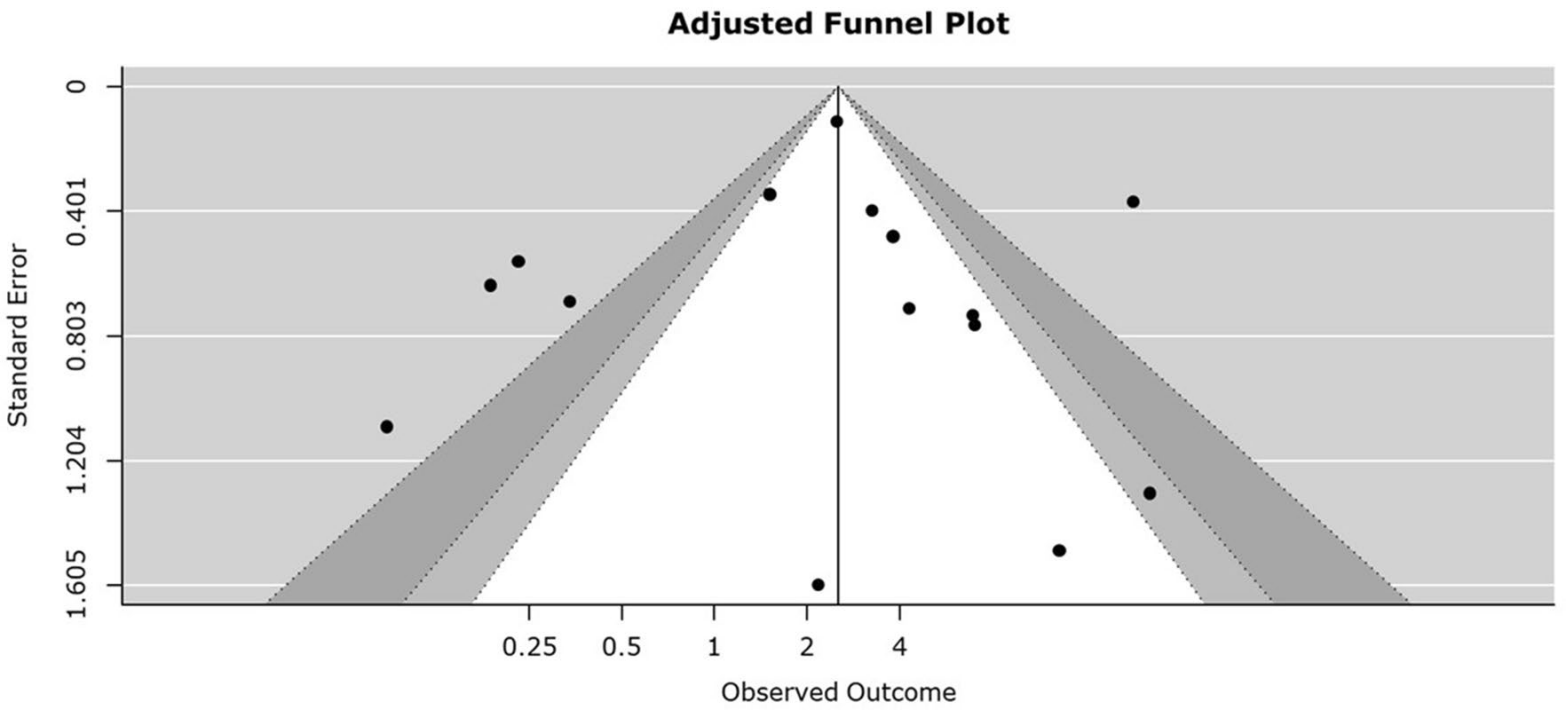
Funnel plot of overall mortality.

In subgroup analysis, Group A had statistically significantly higher mortality in HFOV group with OR 3.18 in fixed model. (Fig. 3) Group B had significantly lower mortality in the HFOV group with OR 0.22 in fixed model. In group C, OR was 1.69 in fixed model, but it was not statistically significant. The funnel plot for overall mortality is reported in Fig. 4.

The funnel plots for overall mortality are shown in Fig. 4.

### The incidence of CLD

**Figure 5:**
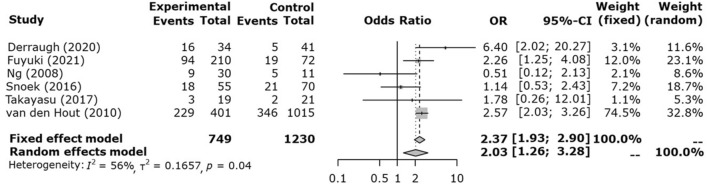
Forest plot of the incidence of chronic lung disease.

Regarding the incidence of CLD, the OR and 95%CI were 2.37 and 1.93–2.90, respectively, for the fixed model. In other words, the incidence of CLD was significantly higher in HFOV (Fig. 5).

The funnel plots for the incidence of CLD are presented in the supplemental materials.

## Discussion

CDH that needs not only various medical supports according to the underlying condition of the patients but also requires surgical treatment of diaphragmatic defect is a complex disease that shows high mortality rate even with modern developed perinatal care. The mortality rate is known to reach 40–100% in low-, middle-income countries whereas reported as 30% in developed countries^2,19^. Careful perinatal management is required, especially for respiratory system, pulmonary HTN, and cardiac dysfunction, which are critical for the patient's survival. The most important point of the respiratory management is to use management that can prevent ventilator-induced lung injury^20^. The introduction of gentle ventilation contributed to the improvement of survival rate. Delayed repair and extracorporeal membrane oxygenation contributed to increase the survival rate by 50% until the early 1990s, and permissive hypercapnia improved the survival rate by an additional 10%^19^. Mechanical ventilation strategies such as HFOV and HFJV have been introduced in clinical practice and are being applied for the unstable CDH patients^21,22^.

This meta-analysis compares the outcome of CMV and HFOV of CDH in over three thousand new-born children with CDH. This study shows that the evidence for the comparison of HFOV and CMV for CDH is scarce. In subgroup analysis, the patients with HFOV showed better survival in group B in which showed chronological analysis of the outcomes of CMV and HFOV.

A pooled result of all enrolled studies for mortality is higher in HFOV than in CMV. (OR: 2.53, 95%CI 2.12–3.01) Since there are heterogeneities regarding the application of HFOV all through the enrolled studies, subgroup analysis was performed. Group A in which applied HFOV in the unstable patients who were initially applied CMV showed statistically significant higher mortality in HFOV (OR: 3.18, 95%CI 2.63–3.84). Group B in which analyzed the outcomes of CMV before the introduction of HFOV and of HFOV after the introduction of HFOV showed statistically significant better survival in HFOV. Group C in which applied either of CMV or HFOV at the timing of the application of MV did not show statistically significant result. The outcome of HFOV could be explained with the characteristics of subgroups. The mortality of group A might be higher in HFOV because the HFOV was selectively applied in poor patients with the use of CMV. More accurate results could be elicited if a randomized controlled study was completed comparing the effectiveness of HFOV and CMV in unstable patients with CDH. Further, those patients in group A where CMV did not treat respiratory acidosis properly may have a benefit from ECMO. HFOV often delays ECMO and therefore survival rate could drop. Early ECMO may have better results^23^. Since group B is a comparison of different periods before and after the introduction of HFOV, other advanced perinatal care other than HFOV may have affected the outcome, resulting in a low mortality rate in HFOV. There are many developments in the care of CDH not only in the area of mechanical ventilation strategy. Surfactant use, fraction of inspired oxygen, the concept of gentle ventilation, the importance of the management of pulmonary hypertension^24^. The improvement in mortality of CDH could also be related improving prenatal care. Also, in the younger era without protocols concerning to standard of care and ventilator settings, cares were not limited as recommended in a standardized protocol^25^. Group C showed no significant difference, which can be explained as follows. The use of HFOV may have no additional benefit compared to CMV in patients who are stable with CMV alone. Unstable patients could benefit with HFOV whereas either HFOV or CMV could offer similar results in relatively stable patients. Of interest a study published in 2016 by Snoek (Group C in this analysis), suggests that HFOV caused overinflation and had a longer ventilation period, which may have caused ventilator-induced lung injury by more tracheal suction. HFOV could have limitations than CMV, which can cause deterioration in vulnerable patients^26^. In the VICI Trial high initial ventilator settings were allowed and there was also a trend to a later initiation of rescue ECMO after initial application of HFOV.^4^ A future study concerning to HFOV should be done with lower settings of HFOV and early ECMO as a rescue treatment.

Chronic lung disease is known to occur in 30–50% of CDH survivors^20^. It is known that the incidence of CLD is higher when ECMO is used and patch repair is performed^27^, but there is insufficient evidence that HFOV reduces CLD incidence^28^. In this meta-analysis, the incidence of CLD was higher in HFOV. Because the number of the enroll studies was small, subgroup analysis like mortality could not be performed. As in mortality, it may have occurred more in HFOV due to baseline characteristic differences between groups more than the difference of the MV modality. Therefore, it seems difficult to reach a clear conclusion.

This study is limited by the heterogeneity of indications for using HFOV or CMV. The evidence from the systematic review is poor due to low level of evidence (a single randomized controlled trial). Also, in further studies risk stratifications should be done by some prenatal information like lung volumes or liver position, or analysis should be done in only isolated CDH patients without other major malformations or syndromes. One study suggested to make a distinction between mild moderate and severe CDH^29^.

On the basis of the poor existing evidence, mortality and the incidence of CLD appear worse after HFOV compared to CMV. Cautious interpretation of this result is needed and only a well-designed prospective study involving large number of patients could address this issue.

## Data Availability

The data generated during this study are available from the corresponding author on reasonable request.
